# Survey on electronic visual field data transfer practices among Japan Glaucoma Society board members

**DOI:** 10.1186/s12886-023-02800-z

**Published:** 2023-02-01

**Authors:** Masaki Tanito, Takeshi Hara, Makoto Aihara

**Affiliations:** 1 Department of Ophthalmology, Shimane University Faculty of Medicine, 89-1 Enya-Cho, Izumo, Japan; 2 Hara Eye Hospital, Utsunomiya, Japan; 3 Department of Ophthalmology, Graduate School of Medicine, University of Tokyo, Tokyo, Japan

**Keywords:** Visual field, Electronic data transfer, Glaucoma, Daily practice, Internet survey

## Abstract

**Background:**

Visual field (VF) testing in combination with a specialized VF analysis software is critical for characterizing and monitoring visual loss in glaucoma. Although performing glaucoma progression analysis requires original VF data rather than printouts or image files, extent of VF data transfer between referring and referred ophthalmologists is unclear. Here, we surveyed glaucoma specialists who belong to the Japan Glaucoma Society (JGS).

**Methods:**

An internet survey of daily practice patterns regarding electronic VF data transfer at the time of glaucoma referrals (referring/referred) was sent to all 50 JGS board members. The survey consisted with 11 questionnaires, and the response rate was 100%.

**Results:**

The respondents included 33 university hospital ophthalmologists (66%) (Q1), and those scattered throughout Japan (Q2). All respondents used Humphrey Visual Filed Analyzer (HFA) (Q3) and at least one of a VF progression analysis software (Q4). Ten respondents (20%) actively transferred electronic VF data, while 40 (80%) did not (Q5). The major reasons for not actively transferring data electronically were that there was no support for data transfer by neighboring (*n* = 26, 65%) and/or own (25, 63%) institutes (Q6). All 40 inactive respondents responded that electronic data transfer is ideal (Q7). All 10 active respondents transferred data using USB flash memory (Q8). Of the 10 active respondents, seven (70%) reported that the percentage of referral letters accompanying electronic VF data in a format that allows for progression analysis from the beginning was less than 25% (Q9). When the referral letters did not accompany the electronic VF data, four (40%) reported that they further requested the data transfer in < 25% of cases (Q10). When the 10 active respondents were requested to transfer data, six (60%) had experienced rejection due to various reasons (Q11).

**Conclusion:**

An internet survey showed that 80% of the JGS board members were not actively transferring VF data mainly because of the absence of a system in place at institutions for sending and receiving data, although they feel that the electronic VF data transfer is ideal. The results provide basic data for future discussions on the promotion of the VF data transfer.

**Supplementary Information:**

The online version contains supplementary material available at 10.1186/s12886-023-02800-z.

## Introduction

Visual field (VF) testing is critical for characterizing and monitoring visual loss in glaucoma and ocular hypertension. For monitoring glaucomatous progression using a specialized software such as Forum (Carl Zeiss Meditec, Jena, Germany) or BeeFiles (BeeLine, Tokyo, Japan), trend- and/or event-based analyses were performed on repeated- and long duration-measured standard automated perimetry testing [1]. General practitioners/ophthalmologists follow many patients with a low-risk glaucoma, and patients requiring further management are sometimes transferred to glaucoma specialists [2]. Performing glaucoma progression analysis requires original VF data rather than printouts or image files (e.g., jpeg and pdf). Therefore, if the referral do not accompany the original electronic VF data, referred ophthalmologists/patients must start collecting VF data for further progression analysis; this scenario is an obvious loss for patients. The same situation also occurs when patients visit other physicians/hospitals because they have relocated. Since it is unclear to what extent of VF data transfer is performed, we surveyed glaucoma specialists who belong to the Japan Glaucoma Society (JGS).

## Subjects and methods

An internet survey of daily practice patterns regarding electronic VF data transfer at the time of glaucoma referrals (referring/referred) was sent to the 50 JGS board members. The institutional review board (IRB) of Shimane University Hospital determined that conducting the survey did not require IRB review/approval because it did not contain patient data. The invitation was e-mailed on August 22, 2022, and the questionnaire was conducted on a web-based survey system (i.e., Google Forms). The 50 members responded to the questionnaire by September 10, 2022 (response rate, 100%) (Table 1). Q1-Q11 and the original responses in English and Japanese are shown in Supplementary File 1.

**Table 1:** Electronic VF data transfer survey results

Q1. Institution	N (%)
University hospital	33 (66)
Eye clinic (≤ 19 beds)	9 (18)
Eye hospital (≥ 20 beds)	5 (10)
General hospital	3 (6)
Q2. Region in Japan	
Tokyo	12 (24)
Kinki	9 (18)
Hokuriku	7 (14)
Kanto	5 (10)
Tokai	5 (10)
Chugoku-Shikoku	5 (10)
Kyusyu	4 (8)
Hokkaido-Tohoku	3 (6)
Q3. Perimetry used	
Humphrey (Carl-Zeiss Meditec, Germany)	50 (100)
Imo (Crewt Medical Systems, Japan)	18 (36)
Kowa (Kowa, Japan)	8 (16)
Octopus (Haag-Streit, Switzerland)	4 (8)
Q4. VF progression analysis software used	
BeeFiles (BeeLine, Japan)	32 (64)
Claio (Findex, Japan)	12 (24)
Perimetry company-provided software (e.g., Guided Progression Analysis, Forum, …)	6 (12)
Q5. When glaucoma patients are transferred (referred/referring), do you actively transfer patients’ electronic VF data in a format that allows progression analysis (not jpeg, pdf, or other data that do not allow progression analysis)?	
No (go to Q6, 7)	40 (80)
Yes (go to Q8-11)	10 (20)
Q6. Why are you not active in electronic VF data transfer?	
No support of data transfer by neighboring institutes	26 (65)
No support of data transfer by own institute	25 (63)
Takes time and effort	13 (33)
Personal data protection	6 (15)
Not required for diagnosis (printout is enough)	4 (10)
Other reason	4 (10)
Q7. Do you think it would be ideal to do electronic data transfer (if the environment is available)?	
Yes	40 (100)
No	0 (0)
Q8. What is your usual method of data transfer?	
USB flash memory	10 (100)
Electric medical record network/cloud system	4 (40)
Floppy disc	3 (30)
Q9. For glaucoma patients, what percentage of referral letters from the referring institutes accompany electronic VF data in a format that allows for progression analysis from the beginning?	
< 25%	7 (70)
25%-50%	2 (20)
50%-75%	0 (0)
≥ 75%	1 (10)
Q10. If the referral letter from the referring institute does not accompany electronic VF data in a format that allows for progression analysis, what percentage requests data transfer further?	
< 25%	4 (40)
25%-50%	4 (40)
50%-75%	0 (0)
≥ 75%	2 (20)
Q11. Have you ever been rejected (or not responded to) a request to provide data to a referring institute?	
Never rejected (including never requested)	4 (40)
Rejected because of "different perimetry equipment"	6 (60)
Rejected because "we don't do data transfers"	4 (40)
Rejected because "we don't know how to transfer data"	4 (40)
Rejected because of "personal data protection"	1 (10)
Rejected because of "no compensation/reimbursement"	1 (10)

## Results

The respondents included 33 university hospital ophthalmologists (66%) (Q1), and those scattered throughout Japan (Q2). All respondents used Humphrey Visual Filed Analyzer (HFA) (Carl-Zeiss Meditec, Jena, Germany) (Q3) and a VF progression analysis software (Q4).

Ten respondents (20%) actively transferred electronic VF data, while 40 (80%) did not (Q5). The major reasons for not actively transferring data electronically were that there was no support for data transfer by neighboring (*n* = 26, 65%) and/or own (25, 63%) institutes, followed by the time and effort required (13, 33%) (Q6).

All 40 inactive respondents responded that electronic data transfer is ideal (Q7). All 10 active respondents transferred data using USB flash memory; four (40%) also transferred data by an electronic medical record network/cloud system, and three (30%) by floppy disc to support old equipment (Q8).

Based on the experience of the 10 active respondents, seven (70%) reported that the percentage of referral letters accompanying electronic VF data in a format that allows for progression analysis from the beginning was less than 25%, two (20%) reported 25-50%, and one reported (10%) ≥ 75% (Q9). When the referral letters did not accompany the electronic VF data, four (40%) reported that they further requested the data transfer in < 25% of cases, four (40%) reported from 25-50%, and two (20%) reported ≥ 75% (Q10). When the 10 active respondents were requested to transfer data, four (40%) had never rejected the request, while six (60%) had experienced rejection due to "different perimetry equipment”, four (40%) reported that "we don't do data transfers”, four (40%) reported "we don't know how to transfer data”, and two (20%) reported other reasons (Q11).

## Discussion

The survey showed that all JGS board members used HFA and at least one type of progression analyses software in daily practice. However, 80% of the members were not actively transferring data at the time of their patients’ transfers, although all respondents thought that data transfer was preferrable.

The main reasons for inactive data transfer were the lack of a system (including absence of the rule and lack of human resources) for sending and receiving data. The free comments from the respondents indicated that, in many hospitals, the use of USB flash memory brought from the outside was prohibited; thus, a data protection policy by the hospital also seemed to be a barrier to VF data transfer. Lack of knowledge and experience by referring physicians/institutes also was a disincentive to data transfer; thus, education about the importance and specific methods of data transfer also seemed to be required. Transport of the data by the patients themselves in data-protected storage media (e.g., integrated circuit card) might solve the transfer problems; hopefully such systems will be available in the future. Building a cloud system might be another direction for the improvement. It is important to note that these future improvements must be accompanied by development of systems for the protection of personal information during personal information exchange, establishing the rules in the facility and inter-facilities, and solving the problems of expenses and appropriate compensation.

The survey was solely conducted among glaucoma specialists in Japan, thus rate of electronic VF data transfer among general ophthalmologists might be even lower than the reported results. Not limited to glaucoma, conditions related to clinical examination and treatment depend on the various social factors including medical laws, health insurance systems, and economic conditions of each region and country in the world. Thus, discussion from a global perspective is also necessary. Coupled with the development of new progression analysis methods [3], VF testing will continue to be important in diagnosing and managing glaucoma [4]. The results provide basic data for future discussions to promote VF data transfer.

## Supplementary Information

**Additional file 1.**

## Data Availability

All the relevant data are contained in this manuscript. Original survey results conducted in Japanese are available as Supplementary File 1.
