# Supplementary material for: Survey on electronic visual field data transfer practices among Japan Glaucoma Society board members
Source: BMC Ophthalmol. 2023 Feb 1;23:45. doi: 10.1186/s12886-023-02800-z (PMC9890677; doi:10.1186/s12886-023-02800-z)
Supplement: Supplementary file 1 — Additional file 1. [file 12886_2023_2800_MOESM1_ESM.pdf]

Survey of daily practice patterns regarding electronic VF data transfer

- Period：2022.8.22～2022.9.10
- Method: Web survey by Google form
- No. of Q：11
- Subjects：50 JGS Board Member
- Respondents: 50（response rate100%）
- Conducted by：

◆Makoto Aihara (JGS President)
◆Takeshi Hara (Hara Eye Hospital)
◆Masaki Tanito (Shimane University Faculty of Medicine)

- Correspondence：mtanito@med.shimane-u.ac.jp

Q1. Institution
N=50

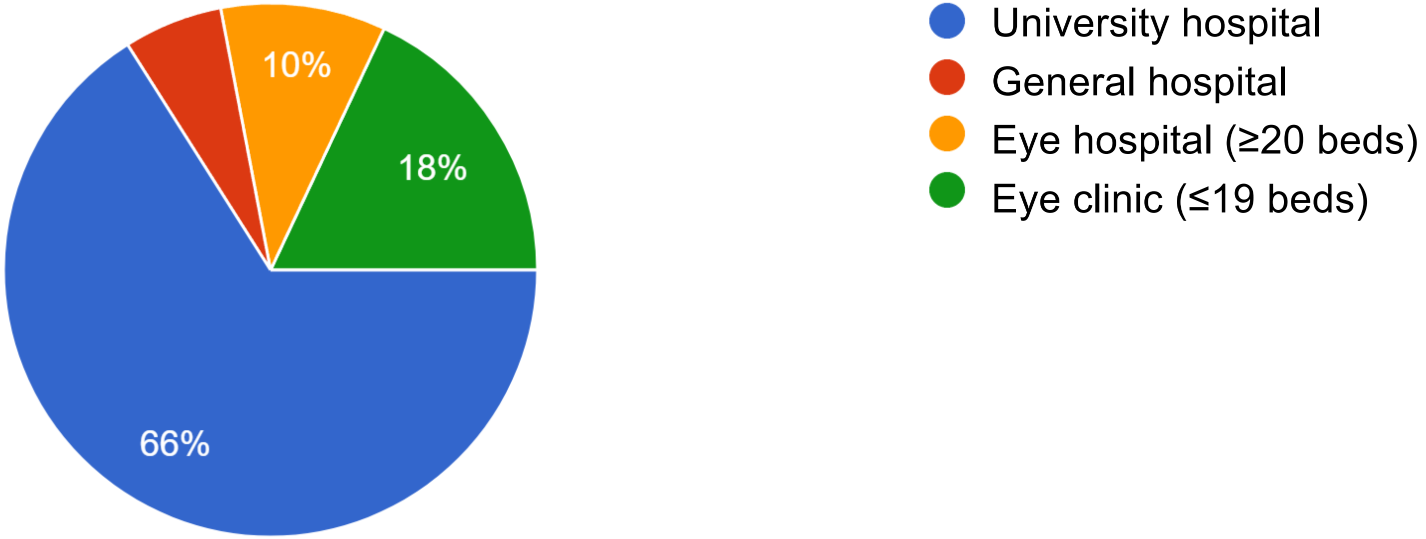

Q2. Region in Japan
N=50

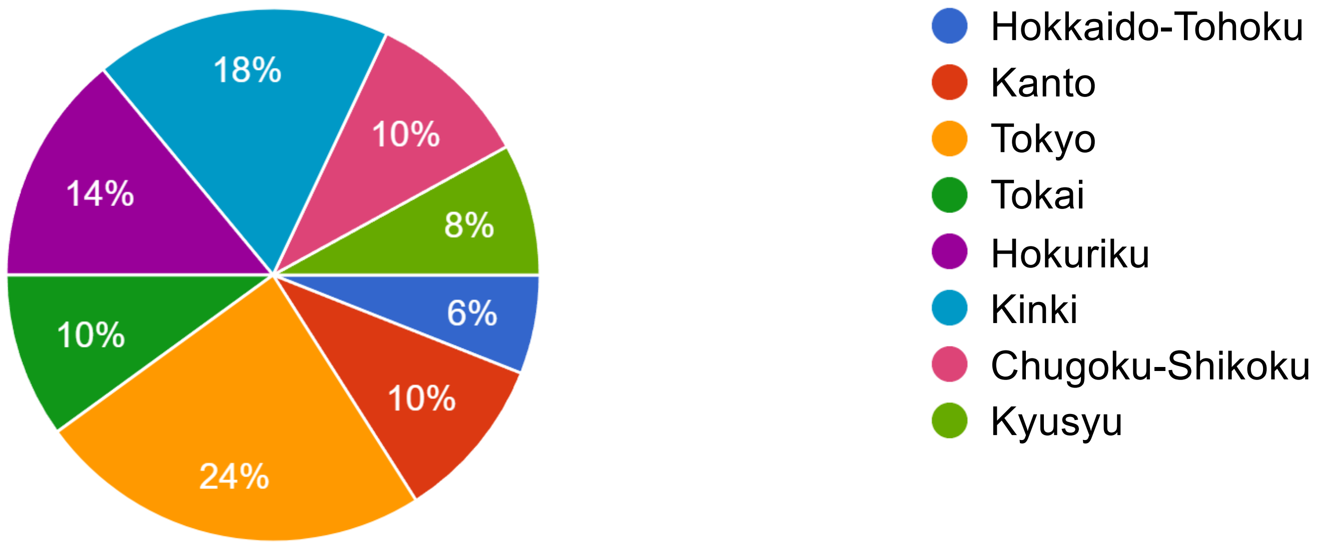

Q3. Perimetry used
N=50

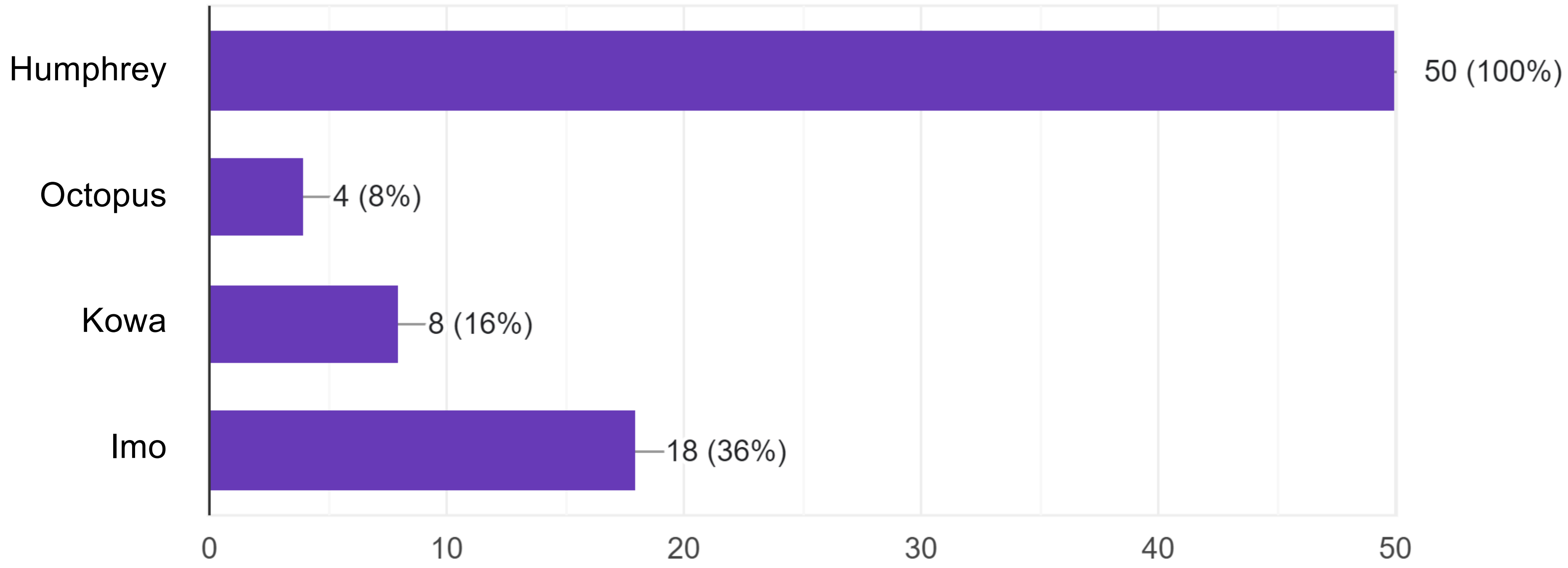

Q4. VF progression analysis software used
N=50

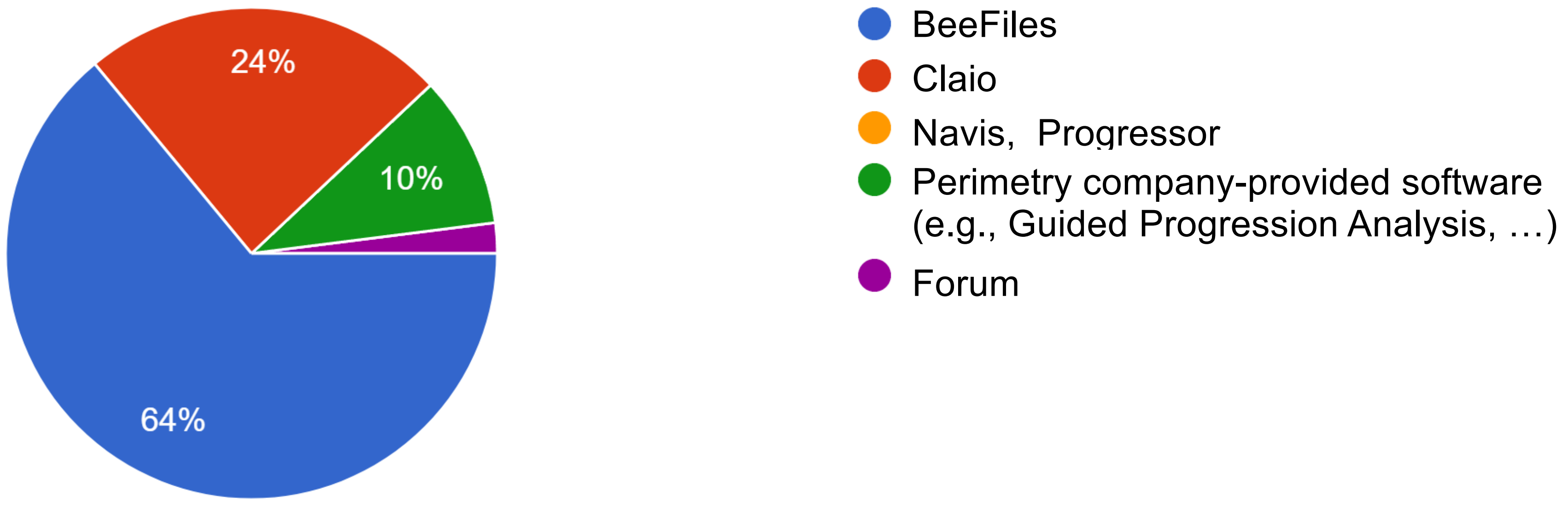

Q5. When glaucoma patients are transferred (referred/referring), do you actively transfer patients' electronic VF data in a format that allows progression analysis (not jpeg, pdf, or other data that do not allow progression analysis)?
N=50

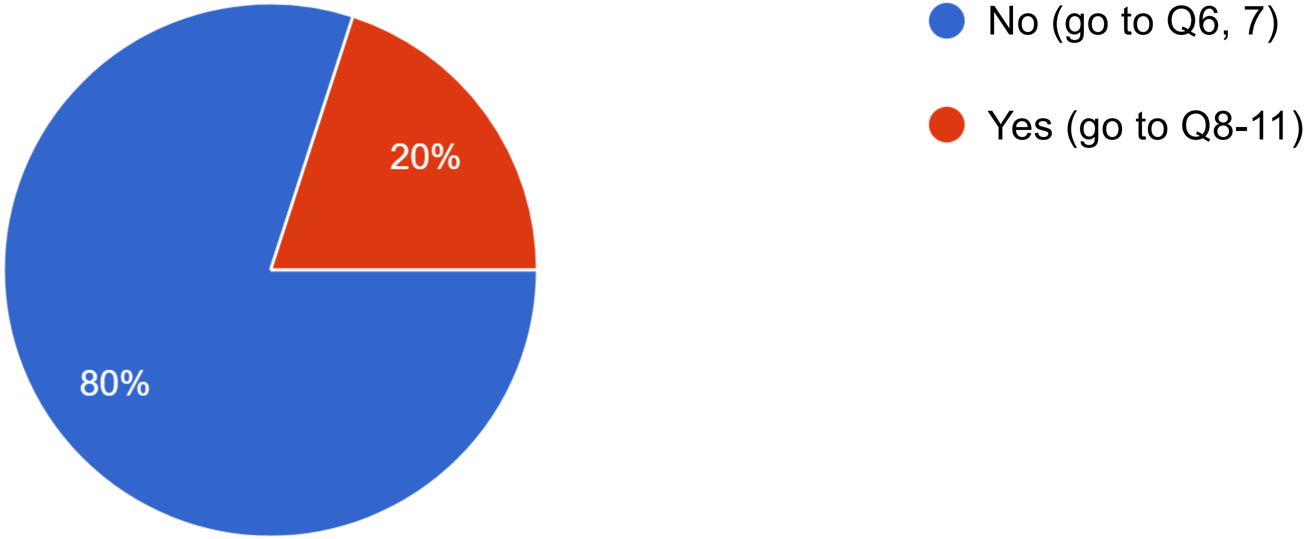

Q6. Why are you not active in electronic VF data transfer?

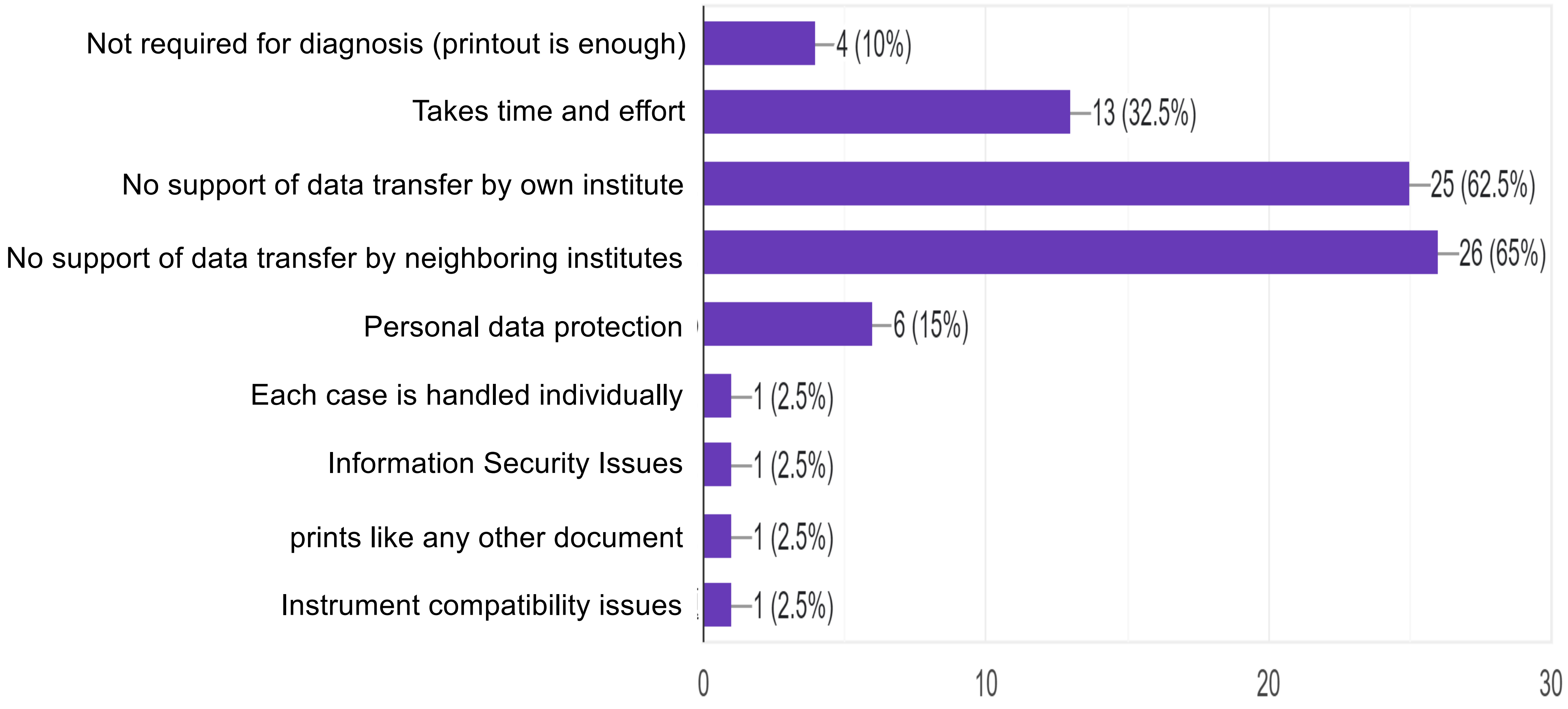

Q7. Do you think it would be ideal to do electronic data transfer (if the environment is available)?

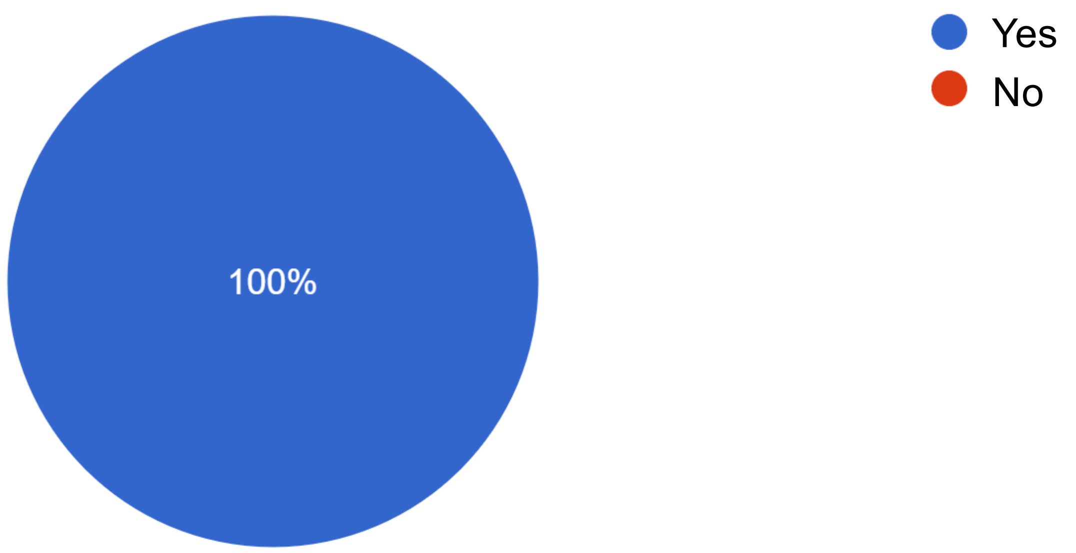

Q8. What is your usual method of data transfer?

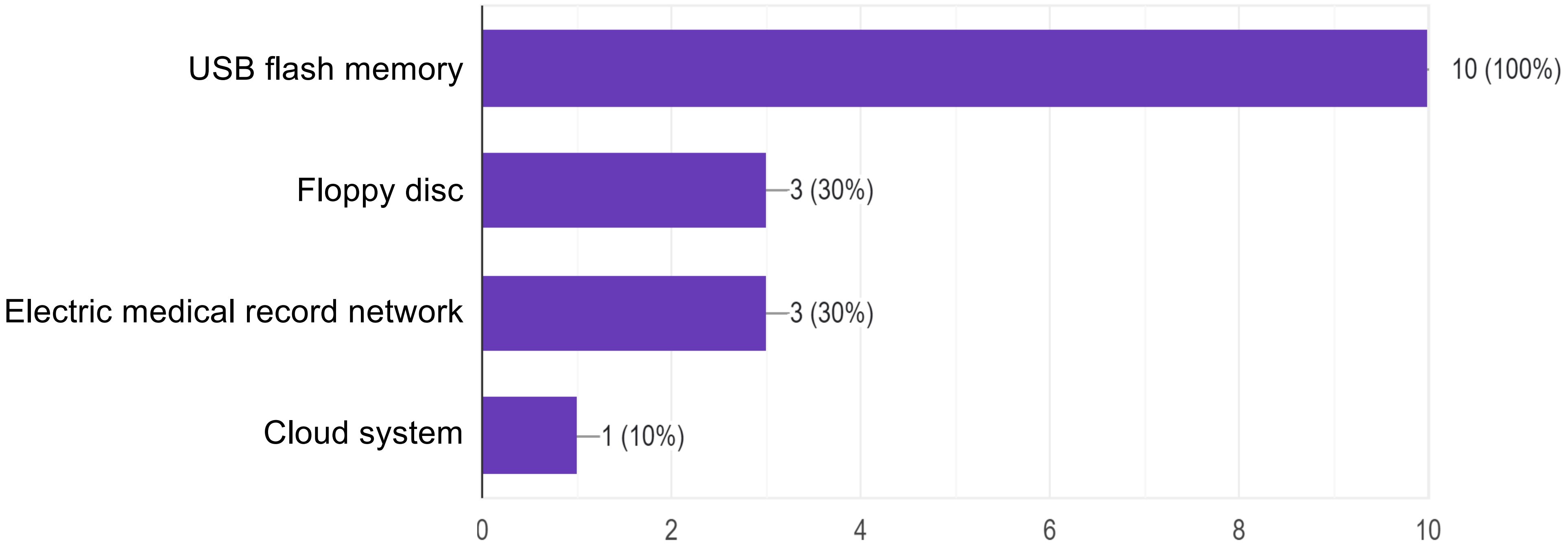

Q9. For glaucoma patients, what percentage of referral letters from the referring institutes accompany electronic VF data in a format that allows for progression analysis from the beginning?
N=10

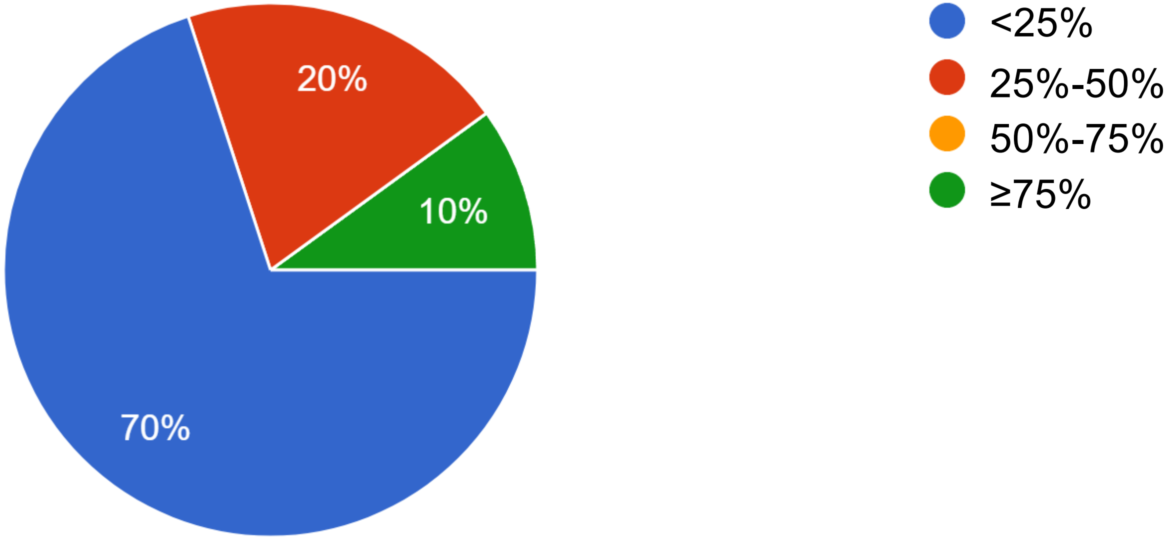

Q10. If the referral letter from the referring institute does not accompany electronic VF data in a format that allows for progression analysis, what percentage requests data transfer further?
N=10

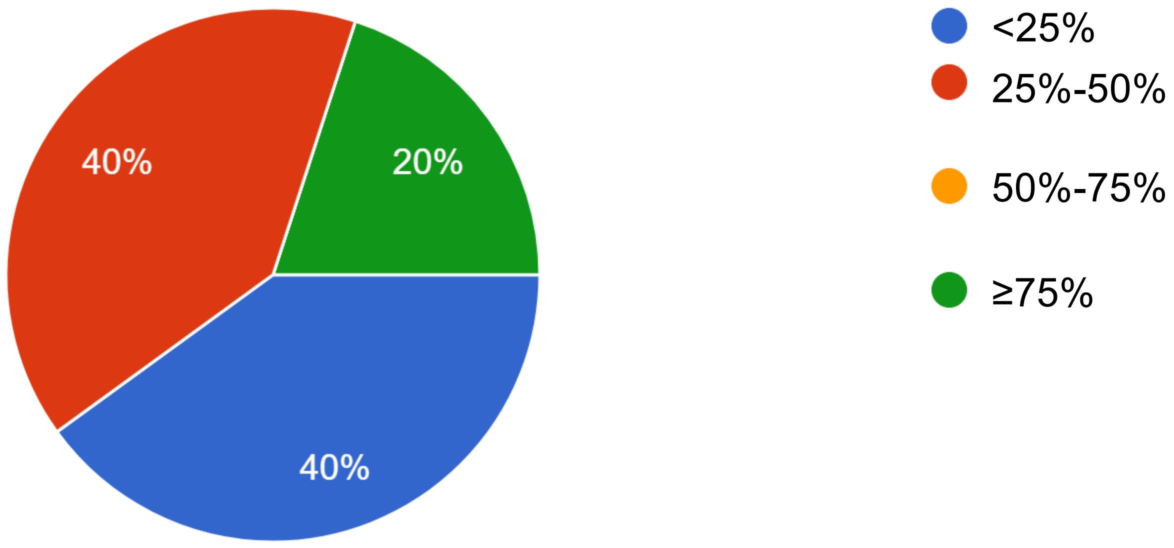

Q11. Have you ever been rejected (or not responded to) a request to provide data to a referring institute?

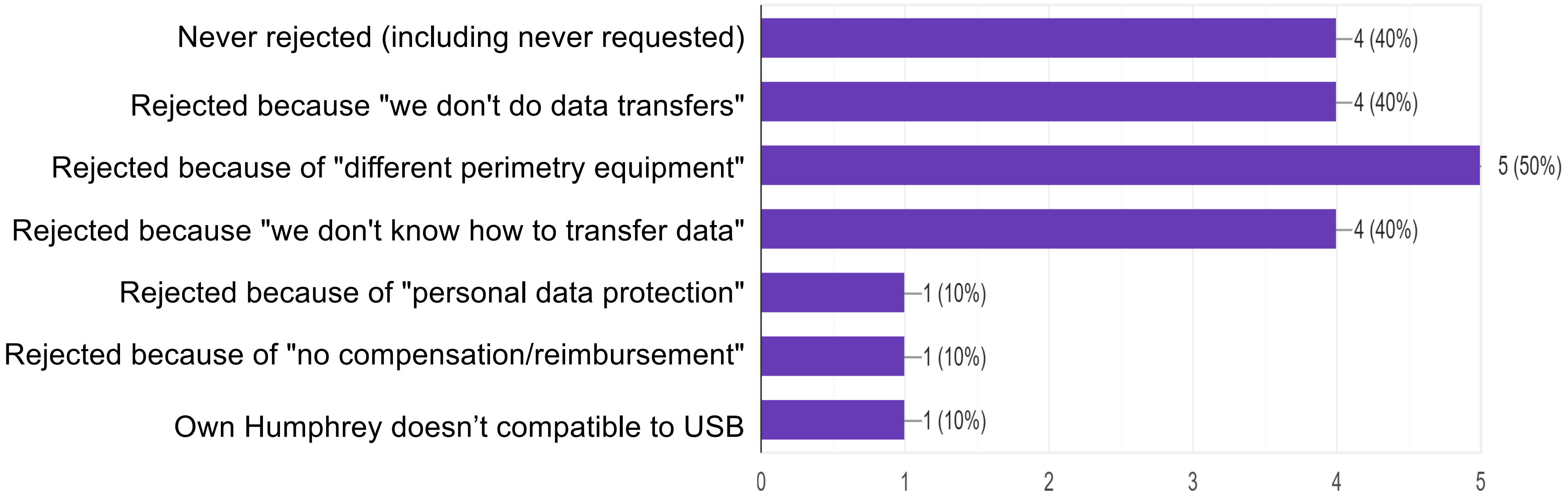

視野データ移送実態調査

• 期間：2022年8月22日～2022年9月10日
• 方法：グーグルフォームによるウェブ回答
• 質問数：11
• 調査対象：日本緑内障学会評議員50名
• 有効回答：50名（回答率100%）
• 実施者：

◆日本緑内障学会理事長・相原 一
◆原眼科病院・原 岳
◆島根大学眼科・谷戸正樹

• 連絡先：mtanito@med.shimane-u.ac.jp

設問１　現在の主な診療環境（回答は１つのみ）

50 件の回答

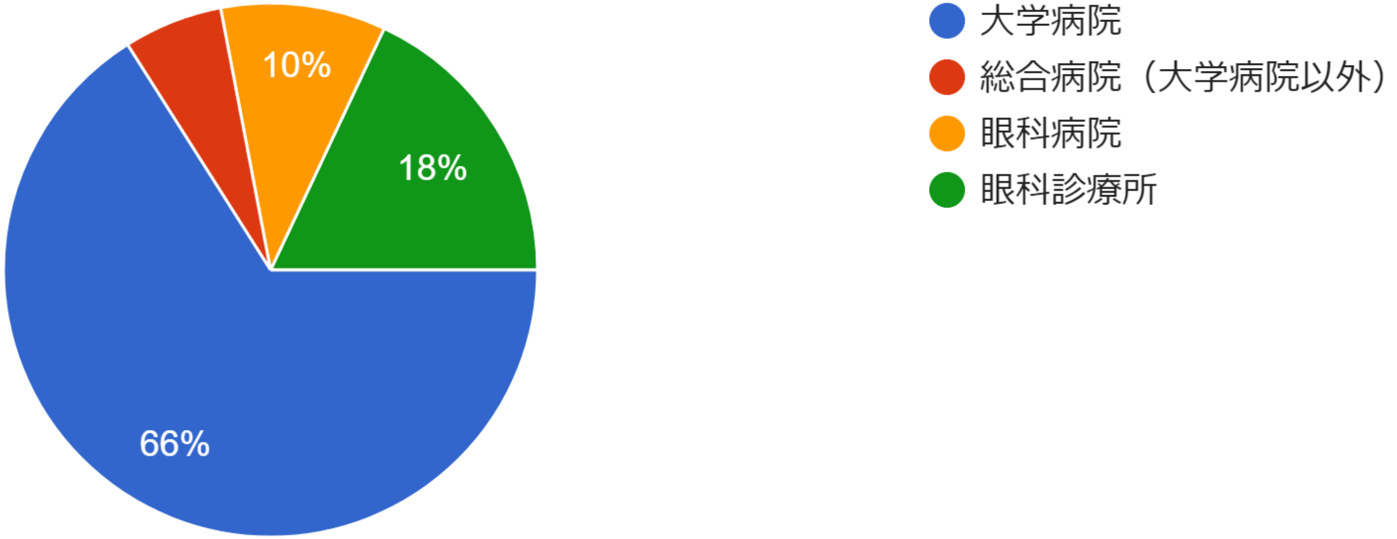

設問２　主に診療を行っている地域

50 件の回答

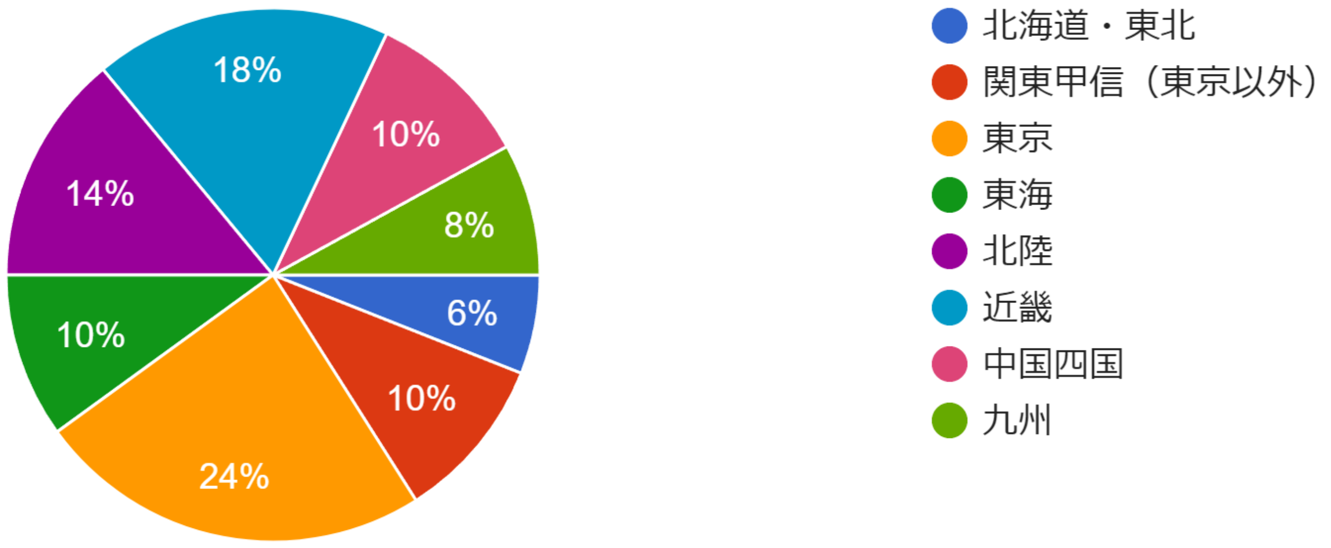

設問３　下記の内，現在使用されている自動視野検査機器（複数回答可）

50 件の回答

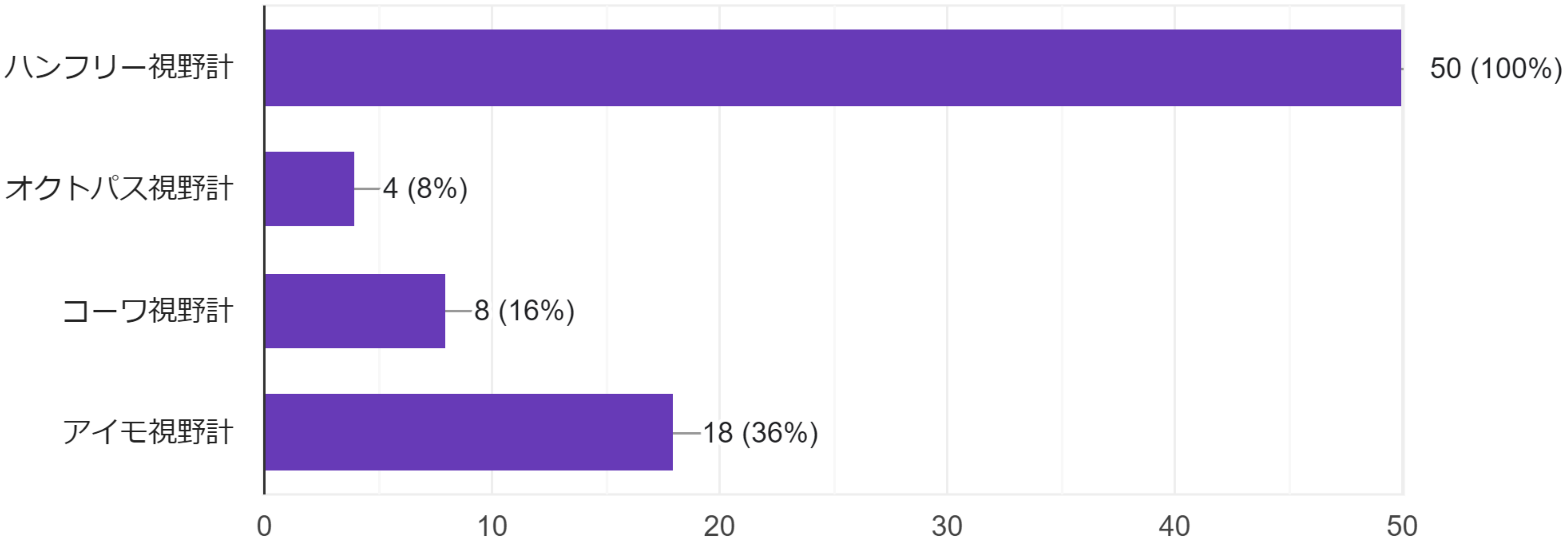

設問４　下記の内，主に使用されている視野進行解析ソフトウェア（回答は１つのみ）

50 件の回答

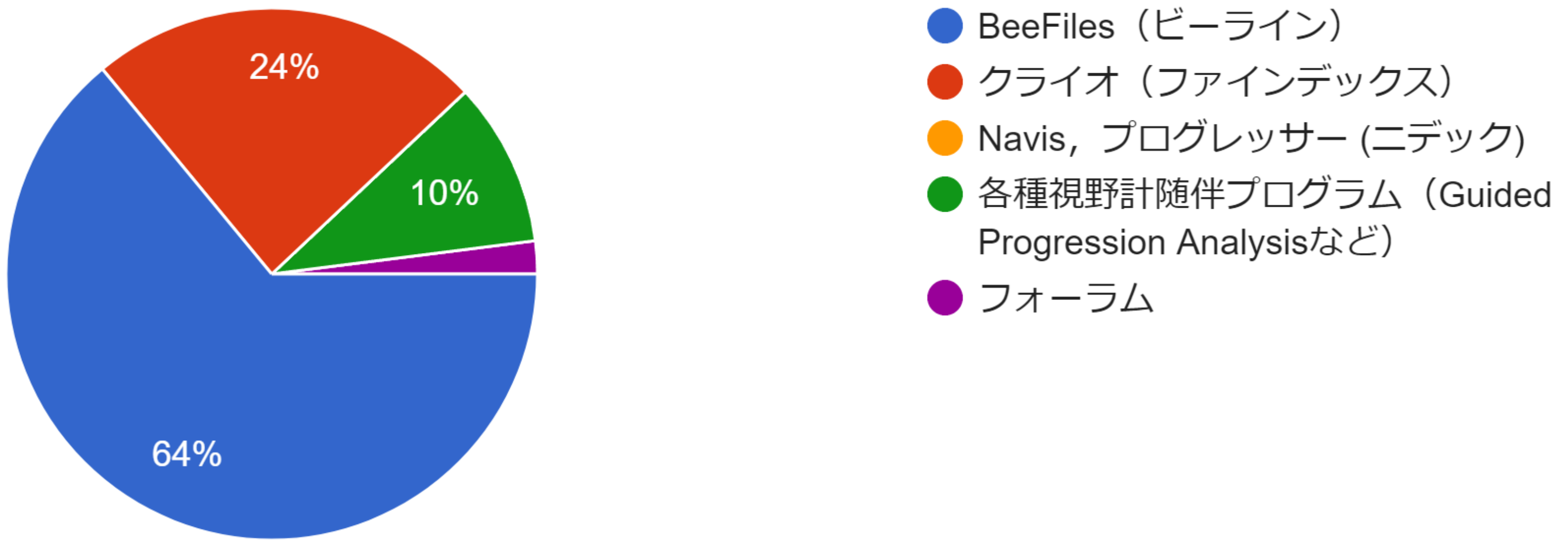

設問５　緑内障の患者さんが転院する（紹介される・する）際に，進行解析可能な形式の視野電子データ（jpeg, pdfなど進行解析不可のデータではなく）の受け渡しを積極的にされていますか？

50 件の回答

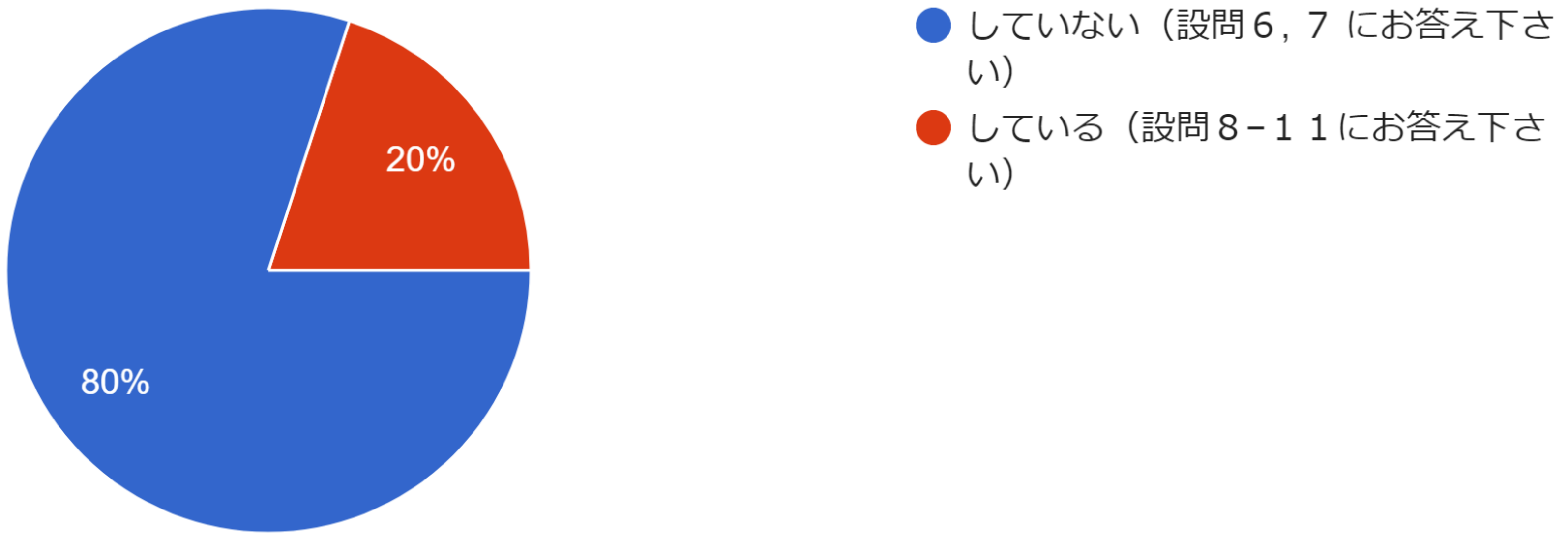

設問６　設問５で，電子データの受け渡しを積極的にしていない方に伺います。していない理由は？
（複数回答可）

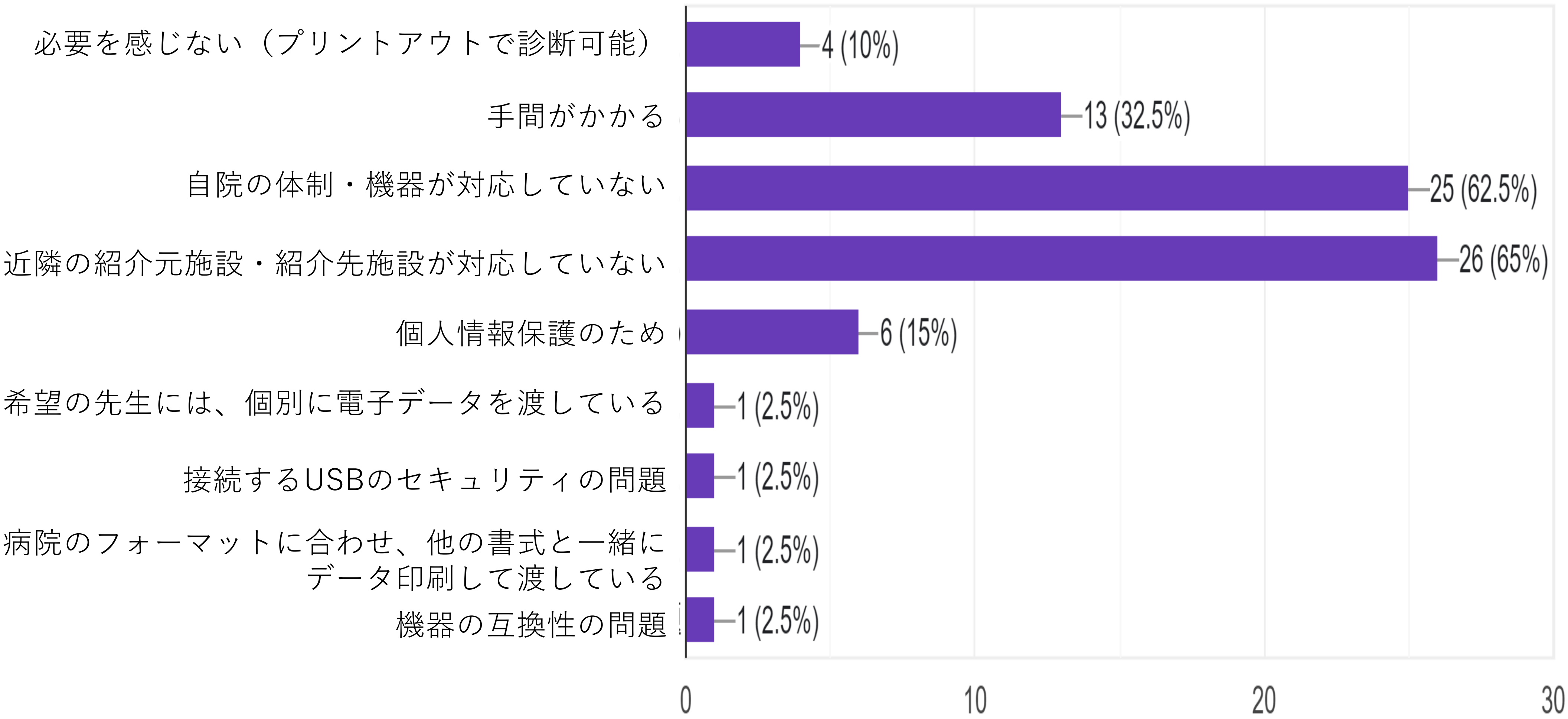

設問７　設問５で, 電子データの受け渡しを積極的に<u>していない</u>方に伺います。電子データの受け渡しは（環境が整えば）あった方が良いと思いますか？（回答は１つのみ）

41 件の回答

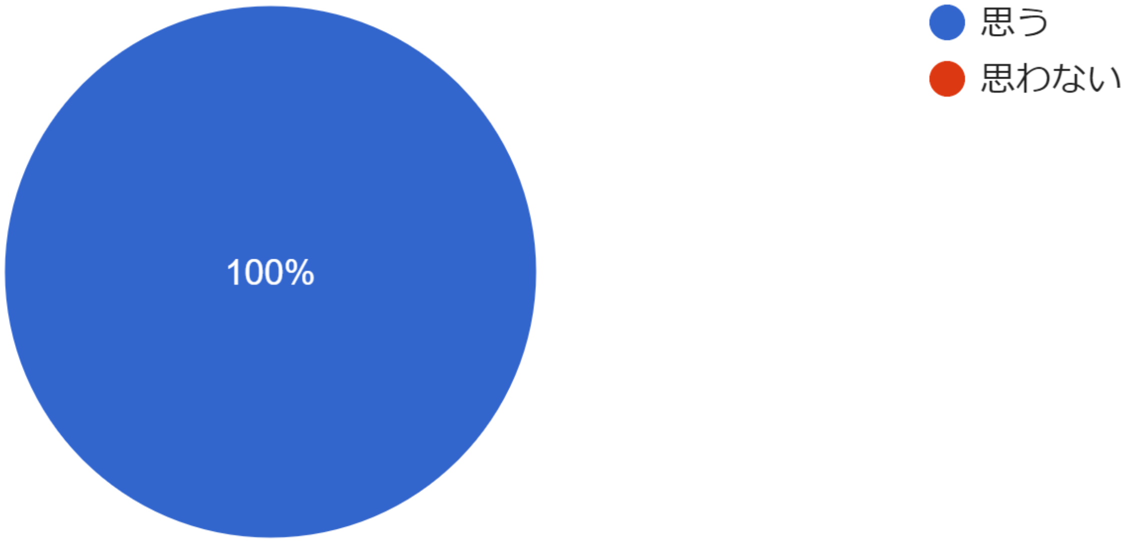

設問８　設問５で，電子データの受け渡しを積極的にしている方に伺います。普段利用しているデータ受け渡しの方法は？（複数回答可）

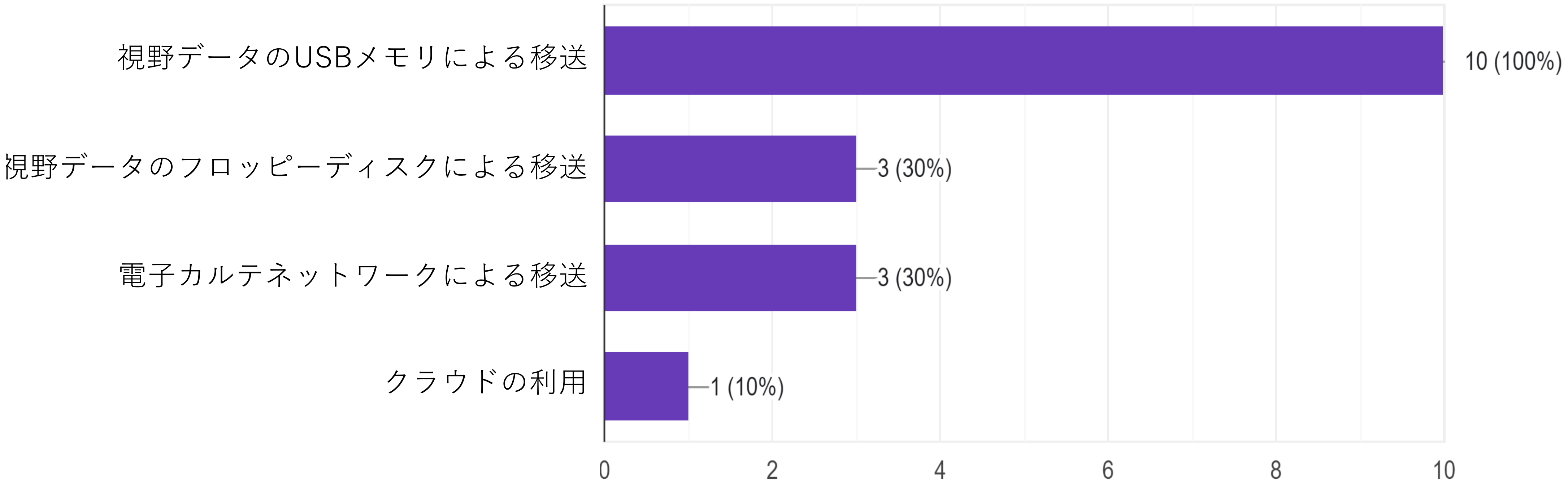

設問９　設問５で，電子データの受け渡しを積極的にしている方に伺います。緑内障患者さんについて，紹介元からの紹介状に進行解析可能な形式の視野電子データが最初から添付されている（送付されている）割合はどのくらいですか？（回答は１つのみ）

10 件の回答

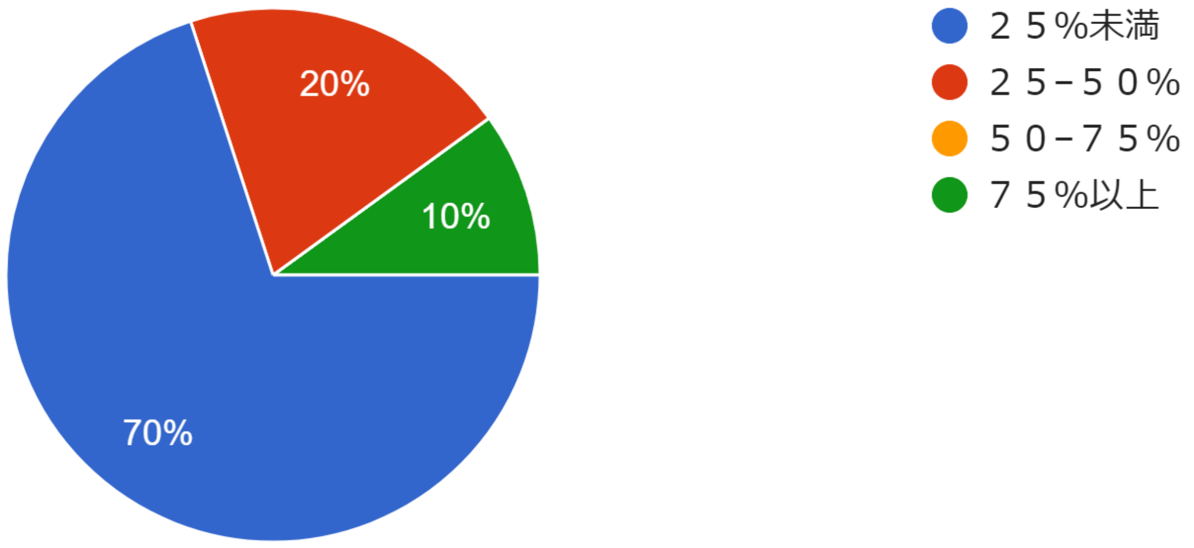

設問１０　設問５で，電子データの受け渡しを積極的にしている方に伺います。紹介元からの紹介状に，進行解析可能な形式の視野電子データが最初から添付されていない（送付されていない）場合，データ提供を依頼しますか？（回答は１つのみ）

10 件の回答

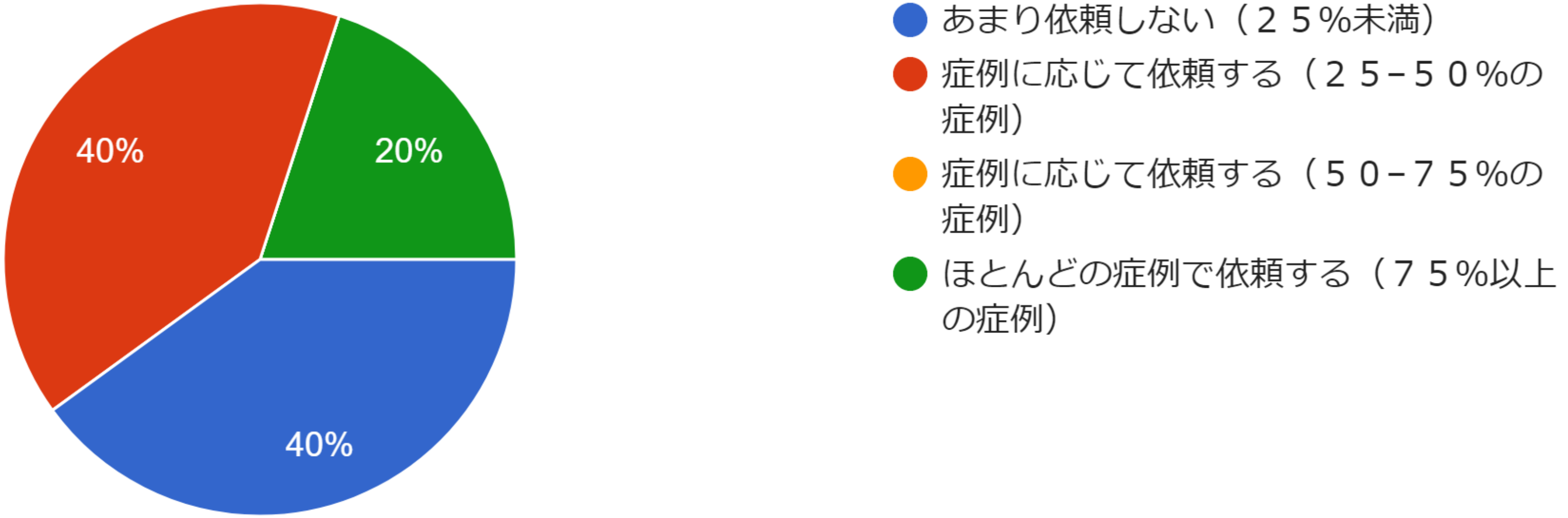

設問１１　設問５で，電子データの受け渡しを積極的にしている方に伺います。紹介元へデータ提供を依頼した際に断られた（対応してもらえなかった）ことがありますか？（複数回答可）

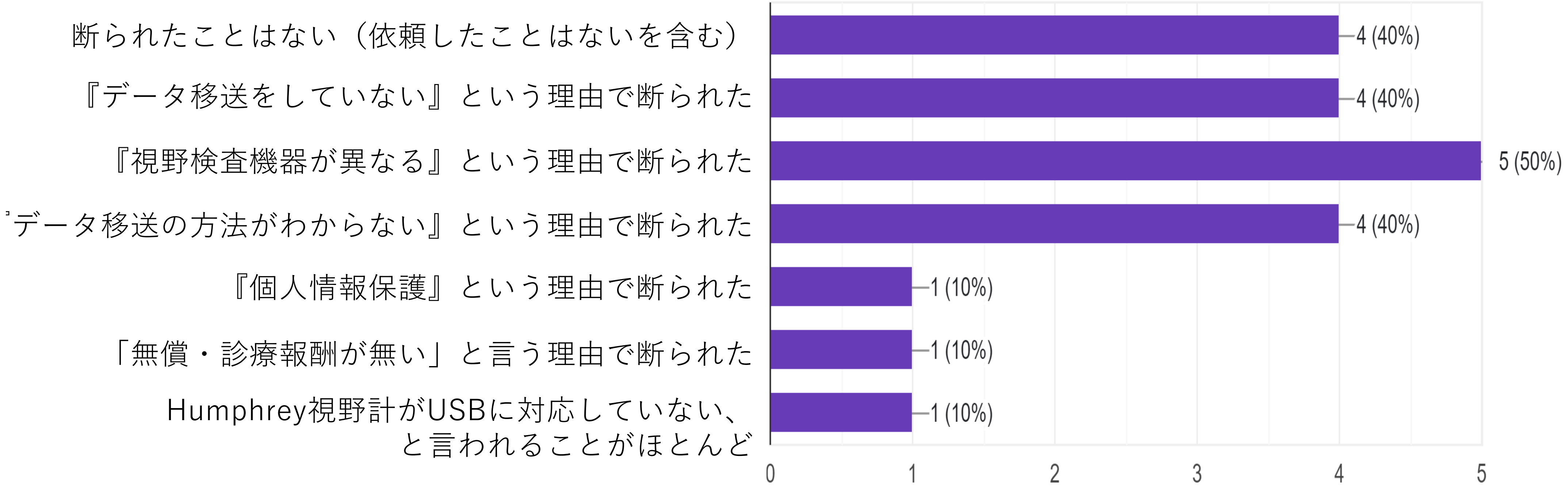

クラウドに患者IDでアクセスし、PW入力で視野データをダウンロードできるようにメーカー側でシステム構築してもらえるよう、学会から働きかけてほしいです。

後から訴訟などに使われる可能性はないでしょうか？

デジタルデータがある程度フォーマットが統一され、患者個人の資産として個人が持ち運びでけいるデータポータビリティが完成すると、長期にわたる緑内障管理では効果を発揮できると思います。

そもそも大学病院、関連病院ではハンフリーですが、紹介元クリニックでは多くの施設がコーワであるため連携の難しさを感じています

大学はHFAを使っているとアナウンスはできても、クリニック側の検査機種を把握することは難しいと感じる。

視野データ継承の推進は今後喫緊の課題と思われます。電子カルテの普及により、今後データ継承が促進されることを強く望みます。

検査施設が異なると、プログラム（24-2or30-2等）、閾値アルゴリズムが異なる、検査環境が異なる、などで厳密な比較は難しい。視野計に取り込むと他施設と自施設の区別がつかないのも問題かと思います。

病院でUSB端子の使用が禁止されており、もちろん外部からのネット接続もできませんので、なかなか難しいです。

転居などで紹介する場合、USBメモリにいれて紹介するのが理想的ですが、病院のセキュリティーの都合で、病院指定のUSBメモリしか使えないのが少々面倒です。でも、患者さんの大事な歴史なので、頑張って紹介先に視野データを持って行けるように頑張っています。
